# A Longitudinal Study of Episodic and Semantic Autobiographical Memory in aMCI and Alzheimer’s Disease Patients

**DOI:** 10.3390/ijerph18136849

**Published:** 2021-06-25

**Authors:** Juan C. Meléndez, Alfonso Pitarque, Iraida Delhom, Elena Real, Mireia Abella, Encarnación Satorres

**Affiliations:** 1 Department of Developmental and Educational Psychology, Faculty of Psychology, University of Valencia, Av. Blasco Ibañez 21, ES 46010 Valencia, Spain; erecom@alumni.uv.es (E.R.); miafer2@alumni.uv.es (M.A.); Encarna.Satorres@uv.es (E.S.); 2 Department of Methodology of the Behavioral Sciences, Faculty of Psychology, University of Valencia, Av. Blasco Ibañez 21, ES 46010 Valencia, Spain; Luis.A.Pitarque@uv.es; 3 Department of Psychology, Faculty of Psychology, Universidad Internacional de Valencia, Pintor Sorolla 21, ES 46002 Valencia, Spain; iraida.delhom@campusviu.es

**Keywords:** autobiographical memory, mild cognitive impairment, Alzheimer’s disease

## Abstract

Background: The main objective of this study was to analyze the evolution of autobiographical memory (both episodic and semantic) in patients with mild cognitive impairment, patients with Alzheimer’s disease, and a healthy control group. We compared these groups at two time points: first, at baseline, and in a follow-up after 18 months. Method: Twenty-six healthy older adults, 17 patients with mild amnestic cognitive impairment, and 16 patients with Alzheimer’s disease, matched on age and educational level, were evaluated at both time points with the Autobiographical Memory Interview. Results: The results showed significant longitudinal deterioration in episodic and semantic autobiographical memory in patients with mild cognitive impairment and in patients with Alzheimer’s disease, but not in healthy older adults. Conclusions: The deterioration of episodic and semantic autobiographical memory in AD is confirmed; however, although the episodic was impaired in aMCI, a pattern that evolved toward deterioration over a period of eighteen months was observed for the semantic autobiographical memory.

## 1. Introduction

Autobiographical memory (AM) is a type of declarative memory that refers to the personal past and allows the recovery of personal semantic data as well as incidental or episodic memories, which means bringing first-person past situations experienced into the present. It has been well established that the normal aging process is generally associated with changes in various cognitive domains, including memory, and memory impairment is the most common cognitive symptom of amnestic mild cognitive impairment (aMCI) and Alzheimer’s disease (AD) [1]. However, knowledge about the progression of AM over time is scarce. The lack of research on this topic is surprising, given that changes in memory are one of the key factors in both normal aging and in cognitive pathologies experienced by older adults. These changes appear from the beginning in the course of neurodegenerative diseases, and they continue to deteriorate with the progression of disease severity [2]. Studying the progression of AM over time is an important line of research that will help us to understand the evolution of AM and the possible changes that occur in this type of memory.

AM is a uniquely human form of memory that moves beyond the recall of experienced events by also integrating with these memories their interpretation and personal evaluations. Autobiographical memories are rich in thoughts, emotions, and evaluations about what happened, and they provide explanatory frameworks that contain human intentions and motivations [3]. AM constitutes our personal history and allows us to build an identity and continuity [4].

AM brings together general knowledge from one’s past (semantic memory) and from specific events (episodic memory) [5]. Semantic memory is objective memory related to accumulated knowledge of the world that has been organized conceptually, reflects our knowledge of the world, and contains generic information acquired in different contexts [6]. Episodic memory refers to the capacity to recall individual events associated with the perceptual and sensory details collected in the context of a specific time and place; the essence of this type of memory is its specificity. The main difference lies in the fact that episodic memories allow us to re-experience the event, whereas semantic memories do not involve the re-experiencing of events although they contain information that includes general knowledge.

Due to the multi-modal nature of AM retrieval, several functional domains are engaged during recollection. Two meta-analyses [7,8] report that, consistent with previous reviews of AM imaging studies, nearly all the studies on AM retrieval reported an activation of the prefrontal cortex, which plays an important role in episodic memory retrieval. In addition, they point out that more than half of the AM imaging studies reported an activation in the medial temporal lobe region (MTL), and especially the hippocampus, a region that the authors identify as a core contributor to the episodic AM network.

Several authors have shown that normal aging affects the nature of episodic AM and reduces access to contextually specific details [4], whereas studies that have examined semantic memory functioning in aging indicate that it can be preserved or even facilitated in older adults [9]. Some authors even point out that older adults, on average, produce fewer episodic details but more semantic information during AM retrieval than younger adults [10].

The results with aMCI patients have shown impaired episodic AM, but contradictory results for semantic AM. At the neuroanatomical level, there is a decrease in the volume of the hippocampus and the entorhinal cortex of the medial temporal lobes of aMCI patients [11]. In fact, when atrophy occurs in these regions, this could be a sensitive predictor of progression from aMCI to AD [12]. Memory performance for autobiographical episodes is impaired; however, aMCI patients produce autobiographical narratives characterized by a decrease in episodic details and an increase in semantic memories [12,13], These results are consistent with studies showing that aMCI patients scored worse on memory of autobiographical incidents than healthy older adults [14,15], but better than AD patients [15]. However, personal semantic memory remains relatively preserved [12] because when the semantic autobiographical memory is not linked to a context, it is not dependent on the hippocampus. This suggests that although aMCI might be associated with impaired remote semantic memory related to impersonal information such as famous faces, it does not appear to affect autobiographical memory for personal semantic information, especially information connected to a personally relevant event [12]. In some studies, no differences were detected between the AM of healthy older adults and that of an aMCI group, although the aMCI group performed better than the AD group. More specifically, AD patients remembered both remote and recent time periods worse than HOC, while the aMCI group differed significantly with HOC only for recent memory [15]; in contrast, other research [16] demonstrated that personal semantic memory is compromised in aMCI patients in comparison with healthy elderly controls.

AM was found to be impaired in AD patients, and semantic retrieval was poorer in AD patients than in healthy controls and aMCI patients [15,17]. In addition, these results were also observed for autobiographical incidents (episodic) [15], but Barnabe only observed differences between AD patients and healthy controls [17]. This worse AM has shown different temporal profiles [4], with better retrieval being observed for old memories than for recent ones in AD when AM is evaluated as a global score (semantic and episodic AM grouped together) [16]. However, when considered alone, episodic memories tend to be impaired regardless of the time interval, whereas old semantic memories tend to be better preserved than recent ones [18]. It should be noted that the relative preservation of old semantic autobiographical memories occurs only for mild AD because with the progression of the disease, these memories also become prone to substantial loss. It can be argued that representations of personally relevant knowledge that became part of semantic memories earlier in life are more strongly integrated in the brain, better consolidated, and therefore, less degraded by AD [19]. The episodic memory findings suggest that the more frequently retrieved autobiographical memories generally become more independent from the hippocampal complex, and might thus be better protected from early hippocampal damage related to AD [20].

Evidence shows a deterioration in autobiographical memory at the onset of the neurodegenerative disease typical of dementia, but not how the deterioration progresses toward more severe symptoms. To our knowledge, few longitudinal studies [2,14,21] have contrasted the evolution of AM impairments, even though it is important to know how the deterioration will advance in order to determine what aspects of episodic and semantic memory will be affected. To discover the evolution of autobiographical memory, we studied groups of healthy older controls, aMCI, and AD for 18 months. We compared the three groups at baseline and in the follow-up—first for episodic autobiographical memory, and second for semantic memory—to discover the differences in their performance. Moreover, the longitudinal evolution of each group was analyzed for each type of memory in order to find out the possible changes after eighteen months.

## 2. Materials and Methods

### 2.1. Participants

Initially, 91 subjects participated in the study, although only 59 completed it; only older adults over 65 years old (range 65–87, average 75.94) who completed the cognitive and neuropsychological assessment at baseline (T1) and follow-up (T2) were selected for this study on AM. Participants were classified into three groups: healthy older controls (HOC; *n* = 26), amnestic Mild Cognitive Impairment (aMCI; *n* = 17), and dementia-type Alzheimer (AD; *n* = 16) (see Table 1 for sociodemographic).

The general inclusion criteria for the study were: age > 65, and no significant asymptomatic neurovascular disease, history of previous symptomatic stroke, medical condition significantly affecting the brain, motor-sensory defects, alcohol or drug abuse/dependence, serious psychiatric symptoms, or depressive symptomatology. Patients in the aMCI group met the diagnostic criteria specified by Petersen [22], and they were at levels 2 and 3 on the Global Deterioration Scale (GDS) [23]. The inclusion criteria for AD were: diagnosis of AD determined by the DSM-V [24] and reaching levels 3 and 4 on the Global Deterioration Scale [23].

Via the use of G*Power (G*Power 3.1.9.7, Düsseldorf, Germany) in computation, a priori statistical power analysis indicated a minimum total sample size of 66 for a power of 0.95 (α = 0.05; 1 − β = 0.95; three groups; 2 measurements, and correlation among repeated measures of 0.5) to detect a medium effect size (*f* = 0.25), in an F test of repeated measures for within–between interaction. Finally, with *n* = 59, a sensitivity statistical power analysis indicated that this design is able to detect a medium effect size of 0.25 (*f* = 0.2628; α = 0.05; 1 − β = 0.95).

### 2.2. Procedure

Clinical diagnosis was the end result of an extensive evaluation, which included medical history and physical and neuropsychological examinations, and was determined by consensus between the neurologists and a neuropsychologists (see Table 1 for neuropsychological data). Instruments for clinical assessment are described below. All participants (or close family members) gave written informed consent for participation in the study. The study was conducted according to the guidelines of the Declaration of Helsinki.

After the baseline assessment, the participants were informed that they would be called for a follow-up evaluation after about 18 months. A total of 91 participants initiated the study, met the inclusion criteria, and were assessed and assigned to the groups. Five HOC refused to participate in the follow-up; in the aMCI group, 13 dropped out of the study—6 refused to participate in the follow-up, and 7 were re-diagnosed with dementia during follow-up; in the AD group, 14 dropped out of the study—4 because of death, 7 due to a worse GDS score, and 3 refused to participate in the follow-up.

### 2.3. Materials

#### 2.3.1. General Cognitive Screening

In addition to the GDS [23] and Center for Epidemiologic Studies-Depression Scale (CES-D) [25], all the participants completed a comprehensive battery of neuropsychological tests assessing the main cognitive domains. The CES-D was developed as a measure of depressive symptomatology in the general population. This scale has 20 elements included in previously validated depression scales. As a cut-off point, 16 is usually used, which indicates the presence of clinically significant symptoms.

The Mini-Mental State Examination (MMSE) [26] was used as an index of global cognitive functioning; the maximum score is 30 points. Language ability was assessed using the Categorical and Phonological fluency subtests of the Revised Barcelona Test (TBR) [27]. Verbal memory (short-term recall and delayed recall) was assessed using the Spain–Complutense Verbal Learning Test (TAVEC) [28], a list of 16 words from four different categories (kinds of fruit, spices, items of clothing, and tools) are presented orally five times to the participants; after each presentation, subjects are assessed on the number of words remembered correctly. Then, after a 20 min period, subjects’ delayed recall is assessed. Attention and working memory were tested using Digit Span forwards and backwards [29]. The copy and delayed recall of the Rey Complex Figure [30] were used as measures of visuospatial construction and non-verbal anterograde memory, respectively.

#### 2.3.2. Assessment of Autobiographical Memory

The Autobiographical Memory Interview (AMI) [31] is a semi-structured interview used to assess memory retrieval in two domains: personal semantic and autobiographical incidents that are considered episodic. Questions about personal semantic content (involving retrieval of personal facts from one’s past life) and autobiographical incidents (involving retrieval of episodes or incidents from one’s past) are chosen to evoke memories from three periods (childhood, early adult life, and recent life).

In the Personal Semantics section, the subject is asked to recall information and the scores vary from 0 to 2 depending on the quality of the memory (better memory, better score). Subjects can obtain a maximum of 21 points in each of the three major periods, and 63 points in the whole test; in this section, the AMI offers alternative questions to facilitate responses in people with different circumstances and contexts. In the Autobiographical Incident section, which evaluates episodic memory, subjects should evoke three incidents per period. The score depends on the descriptive richness of the incident and its specificity in time and place. If the memory specifies the temporal moment and place, it obtains 3 points; if it is not very specific and does not include the time or place, it obtains 2 points; if it is a vague memory, it is awarded 1 point; and finally, if there is no answer or the answer is based on a semantic memory, 0 points are awarded; a maximum of 27 points may be attained in the test. Two independent judges individually assessed the responses to calculate inter-judge reliability; ratings were correlated using Pearson’s correlations and obtained *r* > 0.83, which guarantees reliable correction.

#### 2.3.3. Follow-Up Assessment

Fifty-nine participants underwent a follow-up assessment in which they were re-administered the AMI as well as the neuropsychological tests. The average time between baseline and follow-up was 18.1 months (range 16 to 19; *SD* = 0.78) and did not differ significantly between the groups.

### 2.4. Data Analysis

Two mixed ANOVAs with 3 groups (healthy older control, aMCI, and AD; between subjects) × 2 times (T1 and T2; within subjects) were applied to two types of memory (episodic or semantic). Simple-effects tests were applied to analyze the significant interactions. All analyses were carried out using the SPSS 21 (IBM Corp, Armonk, NY, USA) statistical package.

## 3. Results

**Figure 1:**
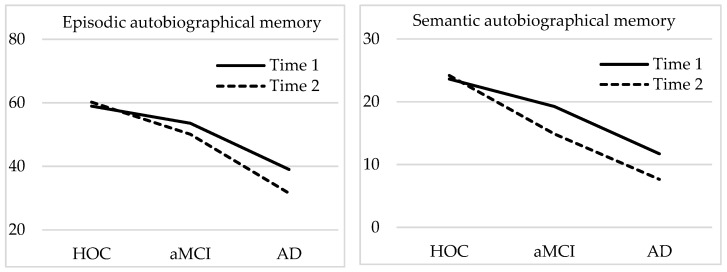
Mean scores for semantic and episodic autobiographical memory obtained at Time 1 and Time 2 by the different study groups.

A mixed ANOVA was performed with three groups (HOC, aMCI, and AD, between-subjects) × 2 times (T1 and T2; within subjects) on the episodic autobiographical memory scores. Results showed significant main effects for both the time (*F* (1, 56) = 29.32; *p* < 0.001; η^2^ = 0.344) and group (*F* (2, 56) = 66.01; *p* < 0.001; η^2^ = 0.213), as well as the interaction (*F* (2, 56) = 13.38; *p* < 0.001; η^2^ = 0.323; see Figure 1). Given the significant interaction, two simple-effects tests were applied. Regarding the differences between groups at each time, the differences were significant at baseline (*F* (2, 56) = 30.88; *p* < 0.001; η^2^ = 0.525), with greater recall in healthy older adults than in aMCI (*p* = 0.010) and AD (*p* < 0.001), and greater recall in aMCI than in AD (*p* < 0.001). At the follow-up assessment, the between-groups comparison also showed significant differences (*F* (2, 56) = 95.25; *p* < 0.001; η^2^ = 0.773), with greater recall in healthy older adults than in aMCI (*p* < 0.001) and AD (*p* < 0.001), and greater recall in aMCI than in AD (*p* < 0.001). Regarding the differences between times for each group, the simple-effects test showed a non-significant effect in the healthy older adult group (*F* (1, 56) = 0.72; *p* = 0.399; η^2^ = 0.013), but a significant decrease in both the aMCI group (*F* (1, 56) = 25.41; *p* < 0.001; η^2^ = 0.312) and the AD group (*F* (1, 56) = 18.31; *p* < 0.001; η^2^ = 0.246).

With regard to the semantic autobiographical memory scores, the mixed ANOVA showed significant main effects for both the time (*F* (1, 56) = 15.38; *p* < 0.001; η^2^ = 0.216) and group (*F* (2, 56) = 54.59; *p* < 0.001; η^2^ = 0.213), and a significant time x group interaction (*F* (2, 56) = 10.19; *p* < 0.001; η^2^ = 0.661; see Figure 1). Given the significant interaction, two simple-effects tests were applied. Regarding the differences between groups in each time, the differences were significant at baseline (*F* (2, 56) = 33.82; *p* < 0.001; η^2^ = 0.547), with no difference between healthy older adults and aMCI, but greater recall in healthy older adults than in AD (*p* < 0.001), and greater recall in aMCI (*p* < 0.001) than in AD (Figure 1). At the follow-up assessment, the between-groups comparison also showed significant differences (*F* (2, 56) = 59.78; *p* < 0.001; η^2^ = 0.547), with greater recall in healthy older adults than in aMCI (*p* < 0.001) and AD (*p* < 0.001), and greater recall in aMCI than in AD (*p* < 0.001). Regarding the differences between times for each group, the simple-effects test showed a non-significant effect in the healthy older adult group (*F* (1, 56) = 1.19; *p* = 0.280; η^2^ = 0.021), but a significant decrease in both the aMCI group (*F* (1, 56) = 5.75; *p* = 0.022; η^2^ = 0.091) and the AD group (*F* (1, 56) = 21.23; *p* < 0.001; η^2^ = 0.275).

## 4. Discussion

The results obtained complement previous findings, confirming that episodic AM was worse when the pathology was more serious; this pattern observed at baseline was maintained at the follow-up. Regarding the longitudinal findings, no significant change was observed in the scores of the healthy controls, but the aMCI and AD subjects showed a significant decrease in their scores on episodic and semantic autobiographical memory.

As the results show, there is a pattern of episodic AM deterioration in the groups of patients as compared to the healthy controls that is maintained in the two time points evaluated, suggesting that when the pathology became more severe, this deterioration was also greater. Some studies have found that episodic memory is impaired with normal aging, finding differences in episodic memory between young and older adults [32,33]. Furthermore, this deterioration in episodic memory is also observed in those studies that compare middle-aged adults with older adults, finding a gradual and progressive deterioration of episodic memory as the person gets older [4,18]. In our study, no deterioration in episodic memory was observed between times in the group of healthy older adults, since the time period was only 18 months. Perhaps, if we could compare their current scores with those obtained years ago, we would have observed this pattern. Regarding the aMCI group, a decrease in episodic recall was observed, although this decrease was not as pronounced as that observed in the AD group. The reduction in episodic autobiographical memory in aMCI could be related to the dysfunction of neocortical structures [12]. In AD, one of the earliest symptoms is a deficit in anterograde episodic memory [34], followed by a retrograde impairment [35]. Moreover, in patients with aMCI and AD, the MTL, particularly the hippocampus and entorhinal cortex, has been found to undergo early volume loss [35], which is associated with a decline in both anterograde and retrograde memory performance. Therefore, the differences observed in episodic AM between the groups of patients could be due to the fact that in aMCI, MTL volume lies between that of healthy elderly people and patients with AD [36].

In semantic AM, two results stand out when comparing the groups. While healthy controls and aMCI at baseline showed differences from the AD group, in the follow-up, differences were obtained between healthy controls and aMCI, and between both groups and AD. The results obtained in aMCI could explain the contradictions found in some studies. Some studies did not detect any impairment in autobiographical semantic memory in healthy older adults [37] and aMCI patients [12]. A possible explanation for the maintenance of semantic memory in the aMCI group could be that the details provided by the participants were not linked to a spatial or temporal context, and therefore did not depend on the hippocampus. Furthermore, this result suggests that neural systems that support these memories, such as the lateral temporal cortex, may remain relatively intact in aMCI [12]. In contrast, other studies demonstrated that personal semantic memory is compromised in aMCI in comparison with healthy elderly controls [16] and pointed to a decline in semantic autobiographical memory for recent memories [15]. As the authors have indicated, these differences in the previous findings may be due to differences in the patient populations studied. When studying the same group at two different time points, it has been observed that both results can make sense, and the deterioration in this type of memory in these patients would be confirmed. This result demonstrates the semantic AM deterioration in the aMCI group eighteen months after the first evaluation and suggests that a decline in autobiographical memory starts as soon as the consolidation of autobiographical information is disturbed by hippocampal damage [15]. Hippocampal volume change gradually intensifies on a continuum, ranging from healthy elderly individuals exhibiting a fairly intact hippocampal structure to aMCI individuals experiencing smaller hippocampal subfields, and finally, to AD patients presenting severe atrophy in all the hippocampal subfields [38]. A possible explanation for our findings is that the initial consolidation of personal semantic facts depends on the hippocampus; therefore, it is also susceptible to early hippocampal damage [15]. In addition, these results are corroborated by the longitudinal analysis of the groups, where a significant deterioration was found in both groups of patients.

Some limitations must be indicated. First, the sizes of the sample groups were unequal, with a difference in the number of participants in the healthy and patient groups. Second, we did not correlate the autobiographical memory function with the subjects’ radiological findings, volumetric assessment of the hippocampus, or functional imaging study. Finally, it should be noted that the instruments for evaluating AM components differ across studies, making it difficult in some cases to compare the results. In this regard, it would be interesting to standardize the evaluation instruments to facilitate comparisons. In addition, it should be noted that when using the Personal Semantics and Autobiographical Incident scores as a combination of the three time points evaluated, it is difficult to compare recent memories and old memories; in future studies, it would be interesting to compare both types of memories in order to know if there is a differential influence on their consolidation.

In summary, the present study showed that autobiographical memory was impaired in patients with AD and aMCI. This deterioration was found mainly in episodic memory in both groups. However, while the deterioration in semantic memory was confirmed in AD, a pattern was observed in the aMCI group that evolved toward deterioration over a period of eighteen months.

## Figures and Tables

**Table 1:** Mean scores (and standard deviations) for demographic and neuropsychological variables obtained at Time 1 and Time 2 by the different study groups.

Variables	a. HOC (*n* = 26)		b. aMCI (*n* = 17)		c. AD (*n* = 16)		Significant Differences (*p* < 0.05)	
	T1	T2	T1	T2	T1	T2	T1	T2
Age	74.53 (4.90)		77.35 (4.76)		77.07 (4.54)		a = b = c	
Gender	10/16		6/11		5/11			
Education	2.78		2.47		2.50		a = b = c	
GDS	1.21	1.1	2.29	2.52	3.35	3.71	a < b < c	a < b < c
CES-D	5.28	5.9	9.01	8.71	9.21	9.14	a = b = c	a = b = c
MMSE	29.04	28.46	21.64	21.35	17.57	17.49	a > b > c	a > b > c
VFTC	23.75	22.64	13.01	11.47	7.64	5.76	a > b > c	a > b > c
VFTP	37.78	38.71	23.41	22.29	14.64	12.35	a > b > c	a > b > c
TAVEC-I	53.28	56.53	27.78	25.35	15.85	13.71	a > b > c	a > b > c
TAVEC-D	12.57	12.67	3.41	3.01	1.07	0.93	a > (b = c)	a > (b = c)
DSF	8.18	8.07	7.46	7.21	5.64	4.98	(a = b) > c	(a = b) > c
DSB	4.96	5.02	3.46	3.25	2.21	1.97	a > (b = c)	a > (b = c)
Rey-I	34.39	34.42	24.37	24.37	16.71	10.35	a > b > c	a > b > c
Rey-D	18.35	19.67	4.53	3.81	0.71	0.42	a > (b = c)	a > (b = c)

HOC: healthy older controls; aMCI: amnesic mild cognitive impairment; AD: Alzheimer’s disease; Gender (male/female); GDS: Global Deterioration Scale; CES-D: Center for Epidemiological Studies–depression scale; MMSE: Mini-Mental State Examination; VFTC: Verbal Fluency Test Categorical; VFTP: Verbal Fluency Test Phonological; TAVEC-I: Spain–Complutense Verbal Learning Test immediate; TAVEC-D: Spain–Complutense Verbal Learning Test delayed; DSF: Digit Span Forward; DSB: Digit Span Backward; Rey-I: Rey Immediate; Rey-D Rey Delayed.

## Data Availability

The data presented in this study are available on request to the authors. The data are not publicly available due to privacy reasons.
